# Clinical Analysis of 36 Cases of Autoimmune Pancreatitis in China

**DOI:** 10.1371/journal.pone.0044808

**Published:** 2012-09-18

**Authors:** Xingang Zhang, Xinpeng Zhang, Wei Li, Li Jiang, Xiaoli Zhang, Yun Guo, Xiaofei Wang

**Affiliations:** 1 Department of Rheumatology, Shengjing Hospital of China Medical University, Shenyang, Liaoning Province, China; 2 Department of Neurosurgery, The Fourth People’s Hospital of Shenyang City, Shenyang, Liaoning Province, China; 3 China Editorial Department of Chinese Pediatric Emergency Medicine Shenyang, Liaoning Province, China; Technische Universität München, Germany

## Abstract

**Background:**

To improve the early identification of autoimmune pancreatitis in China by a retrospective analysis of clinical data from AIP patients.

**Methodology/Principal Findings:**

The analysis included 36 patients admitted by the surgery department of our hospital from January 2003 to October 2011 whose postoperative pathological confirmations were consistent with the histological criteria of Honolulu Consensus Document. The clinical phenotypes associated with the histopathologic patterns of LPSP and IDCP were referred to as type 1 and type 2 of AIP, respectively. A retrospective analysis of clinical features, serological data, pathological findings and imageological records was performed in line with the subtypes of AIP. Type 1 showing a sex predilection (males) was commonly more dominant than type 2 in all AIP. Type 2 without a gender predilection was, on average, a decade younger than type 1. Type 1 was inferior to type 2 in ALT, ALP and γ-GT with statistical significance (*P = *0.044, 0.025 and 0.013). Type 1 was inferior to type 2 in AST with difference close to statistical significance (*P = *0.072). Histopathology revealed frequent lymphoplasmacytic infiltration with less frequent infiltration of neutrophils, eosinophils and fibroblasts. Diffuse and intensive interstitial fibrosis could be seen. The changes of pancreatic head were more frequently seen in type 2 than in type 1 (*P* = 0.05). Plasma cells staining of IgG4 at a density of over 30 or more cells per high-power field appeared to be a specific finding in China with type 1. Imageology found a diffusely or focally enlarged pancreas, most frequently a mass or enlargement in the pancreatic head, characteristic capsule-like rim, calcification or pancreatic calculus and cystic degeneration.

**Conclusions/Significance:**

AIP is a unique type of chronic pancreatitis and has distinctive serological, pathological and imageological characteristics, which should be used for differentiation from pancreatic cancer.

## Introduction

Autoimmune pancreatitis (AIP) is a type of chronic pancreas-specific inflammation mediated by an autoimmune inflammatory reaction. It was first reported by Sarles et al [1] in 1961. Then in 1995, Yoshida et al [2] proposed the concept of AIP that was gradually, widely accepted. Before AIP was recognized as a clinical entity, only 2% to 3% of patients undergoing resection for suspected pancreatic cancer had AIP [3]. In North America, about 2.5% of patients with a preoperative diagnosis of pancreatic cancer are diagnosed with AIP postoperatively. Among chronic pancreatitis (CP) cases, the prevalence of AIP is 5%–6% [4]. Over the past decade, there has been increasing awareness and better understanding of AIP. Many different diagnostic criteria for AIP have been reported from Japan, Korea, Europe, and North America in the past decade [3]. However, Jae Bock Chung considered that there were some limitations of each criterion to satisfy every case of AIP in a consensus meeting on AIP held in Seoul in 2007. AIP was not paid enough attention in the past in China, and the serological and histological testing on IgG4 levels were performed with delay in China. Thus the research about AIP in China laid behind. In retrospect, the diagnostic features of AIP were not well known or recognized in our early experience, and prospectively as AIP became increasingly recognized in China, most of the Chinese patients would be reported in the future. We are eager to clarify the characteristics of AIP in China on basis of studying the current leader experience and achievements. In this study, the clinical data from 36 AIP patients were analyzed retrospectively to improve early differentiation, early diagnosis and early treatment of AIP in China.

## Results

Of 36 cases, 29 cases were included in LPSP (type 1), and 7 cases were included in IDCP (type 2).

### Demographics

These 36 AIP patients included 29 males (25 in LPSP; 4 in IDCP) and 7 females (4 in LPSP; 3 in IDCP), with ratio of 4.1∶1 (ratio of 6.25∶1 in LPSP; ratio of 1.33∶1 in IDCP) and had a mean age of 53.56 years (range, 18–84 years; average, 55.7 years in LPSP and 44.9 years in IDCP); 27 cases were ≥45 years (24 in LPSP; 3 in IDCP). There were no deaths.

### Clinical Features

Initial symptoms included 18 cases (16/29 [55.2%] in LPSP; 2/7[28.6%] in IDCP; *P* = 0.402) with upper abdominal pain, 15 cases (11/29[37.9%] in LPSP; 4/7[57.1%] in IDCP; *P* = 0.418) with jaundice, 5 cases (4/29[13.8%] in LPSP; 1/7[14.3%] in IDCP; *P* = 1) with yellow urine, 4 cases (3/29[10.3%] in LPSP; 1/7[14.3%] in IDCP; *P* = 1) with upper abdominal discomfort, 4 cases (3/29[10.3%] in LPSP; 1/7[14.3%] in IDCP; *P* = 1) with abdominal distension, 2 cases in LPSP with anorexia, 2 cases in LPSP with emaciation, 2 cases in LPSP with weakness, 2 cases in LPSP with increased bilirubin (BIL) occasionally found by physical examination, 1 case in LPSP with periumbilical pain and 1 case in IDCP with waist and back discomfort. Main complications were chronic cholecystitis of 17 cases (12/29[41.4%] in LPSP; 5/7[71.4%] in IDCP; *P* = 0.219); diabetes mellitus of 6 cases (6/29[20.7%] in LPSP); right bundle branch block (RBBB[complete RBBB of 5 cases and incomplete RBBB of 1 case]) of 6 cases (6/29[20.7%] in LPSP); hepatic cirrhosis of 4 cases (3/29[10.3%] in LPSP; 1/7[14.3%] in IDCP; *P* = 1); calculus of the bile duct of 3 cases (3/29[10.3%] in LPSP); pleural effusion of 3 cases (3/29[10.3%] in LPSP); peritoneal effusion of 3 cases (3/29[10.3%] in LPSP); duodenal papillitis of 3 cases (2/29[6.9%] in LPSP; 1/7[14.3%] in IDCP; *P* = 0.488); myocardial ischemia of 3 cases (3/29[10.3%] in LPSP); and renal insufficiency of 2 cases (2/29[6.9%] in LPSP). A loss of body weight was found in 19 cases with a mean loss of 6.58 kg (range, 2–15 kg) (15/29[51.7%] in LPSP; 4/7[57.1%] in IDCP; *P* = 1).

### Serological Data

The tumor marker CA19-9 was increased, >37 U/ml (range, 62.45–859.11 U/ml; average,55.8 U/ml in LPSP and 178.8 U/ml in IDCP; *P* = 0.414), in 10(35.7%) of the 28 tested cases. The tumor marker CEA values were all within the normal range (<5 U/ml) in the 25 tested cases. The diastase was increased, >129 U/L (range, 141–1312 U/L; average, 218.4 U/L in LPSP and 115.3 U/L in IDCP; *P* = 0.298), in 9(45%) of the 20 tested cases. The lipase was increased, >51 U/L (range, 136.9–701 U/L; average, 357.9 U/L in LPSP and 241 U/L in IDCP; *P* = 0.827), in 7(87.5%) of the 8 tested cases. Hepatic function tests were performed in 36 cases as follows. Total bilirubin (TBIL) was increased, >20.5 umol/L (range, 21.3–213.8 umol/L; average, 67.9 umol/L in LPSP and 60.7 umol/L in IDCP; *P* = 0.804]), in 23 (63.9%) cases. Direct bilirubin (DBIL) was increased, >8.6 umol/L (range, 9.8–171.6 umol/L; average, 48.4 umol/L in LPSP and 38.8 umol/L in IDCP; *P* = 0.606), in 22 (61.1%) cases. Indirect bilirubin (IBIL) was increased, >11.9 umol/L (range, 13.6–58.8 umol/L; average, 17.5 umol/L in LPSP and 22 umol/L in IDCP; *P* = 0.518), in 18(50%) cases. Alanine aminotransferase (ALT) was increased, >40 U/L (range, 48–712 U/L; average, 160.1 U/L in LPSP and 332.1 U/L in IDCP; *P* = 0.044), in 25(69.4%) cases, and aspartate aminotransferase (AST) was increased, >34 U/L (range, 35–466 U/L; average, 102.2 U/L in LPSP and 165.9 U/L in IDCP; *P* = 0.072), in 27(75%) cases. However, alkaline phosphatase (ALP) and γ-glutamyltranspeptidase (γ-GT) were evaluated in 34 cases. ALP was increased, >150 U/L (range, 165–1619 U/L; average, 267.4 U/L in LPSP and 593.4 U/L in IDCP; *P* = 0.025), in 22(64.7%) cases and γ-GT was increased, >64 U/L (range, 151–2452 U/L; average, 503.7 U/L in LPSP and 1043.7 U/L in IDCP; *P* = 0.013), in 27(79.4%) cases. The antinuclear antibody (ANA) series of assays was only performed for 1 case in IDCP, the anti-Ro-52 antibody was positive and the antibodies of anti-SSA and anti-SSB were negative. The anti-mitochondrial antibody (AMA) was negative in another 1 evaluated case with LPSP. The IgG level was tested, >15.15 g/L (17.3 g/L and 16.7 g/L), in only 2 cases with LPSP.

### Pathological Findings

Because of the fact that primary pathological diagnosis for AIP in our study was the most fundamental basis, all 36 patients with pathological confirmations had resected specimens. Most specimens were yellow-white, gray-yellow or gray-white, with a fragile or firm texture. There were 9 cases (5/29[17.2%] in LPSP; 4/7[57.1%] in IDCP; *P* = 0.05) with a mass in pancreatic head lesions (1 case with cystic degeneration; 1 case with focal calcifications and 2 cases with nodular enlargement). Four cases (3/29[10.3%] in LPSP; 1/7[14.3%] in IDCP; *P* = 1) had masses in pancreatic body and tail lesions (closely adherent to spleen for 3 cases). Three specimens (3/29[10.3%] in LPSP) were found with cystic degeneration in pancreatic body lesions (1 case with multiple cysts and 2 cases with a single cyst). Three cases (3/29[10.3%] in LPSP) showed fatty nodules in peripancreatic lesions. Only 1 case (1/29[3.4%] in LPSP) was observed with calcification in pancreatic body lesions, and another case (1/29[3.4%] in LPSP) with nodules in pancreatic capsular lesions.

**Figure 1:**
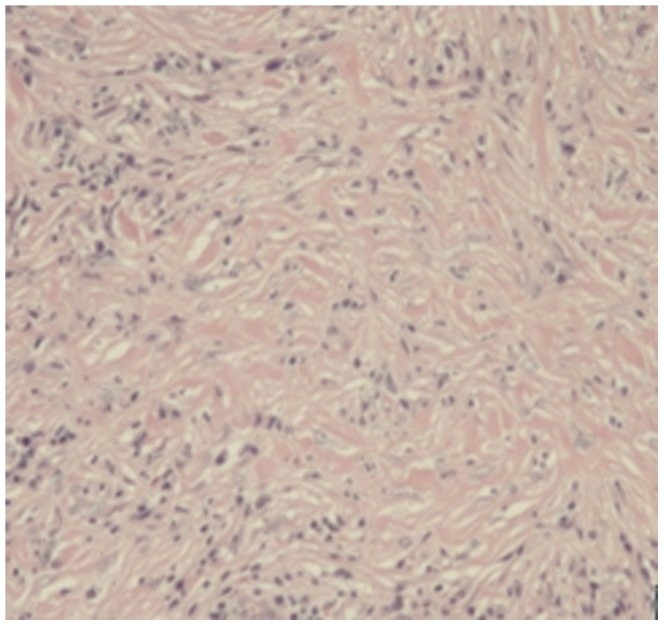
Storiform fibrosis: short collagen bands are randomly interlacing in every direction. (×100).

**Figure 2:**
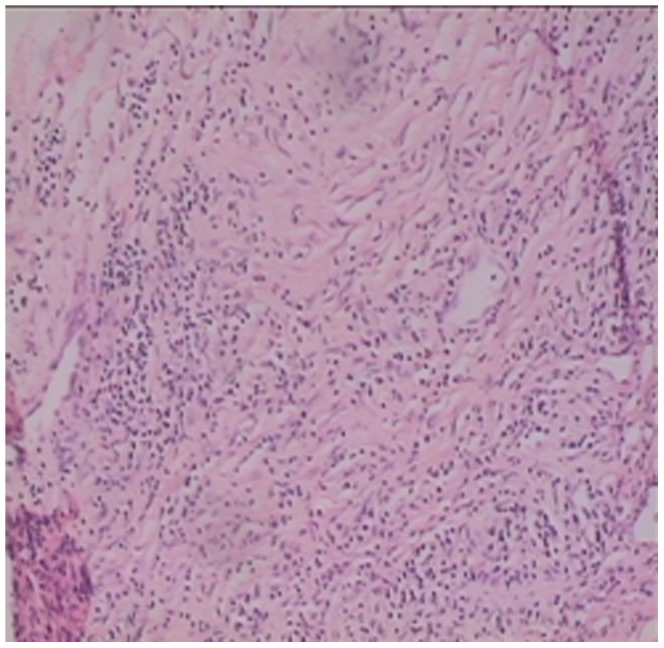
Diffuse and dense lymphoplasmacytic infiltration and marked fibrosis. (×100).

**Figure 3:**
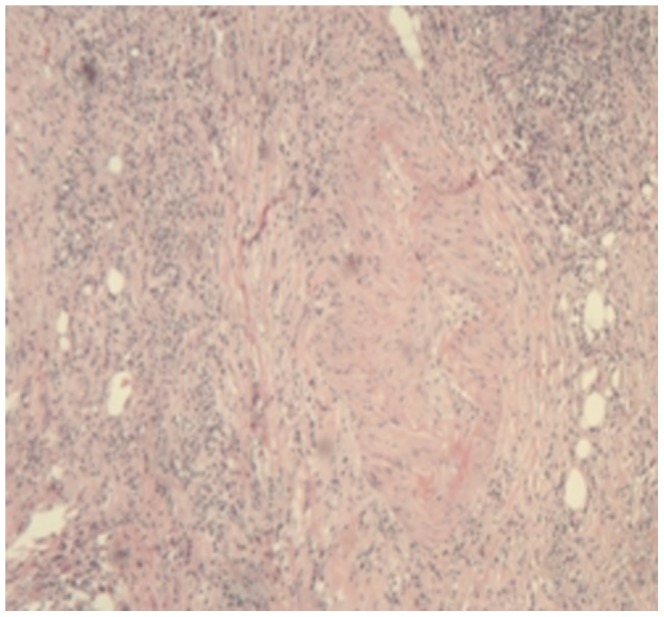
Obstructive Phlebitis: the vein has been completely obliterated by the dense inflammation. (×40).

**Figure 4:**
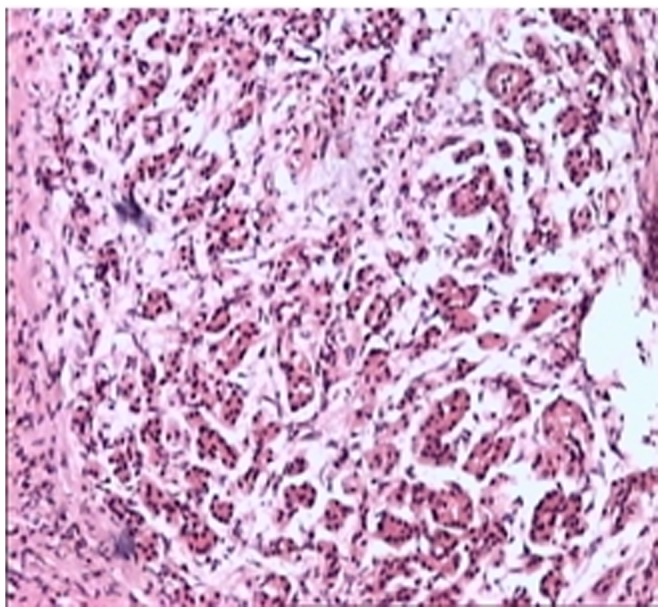
Partial gland adveolus atrophy and dilation of pancreatic duct. (×100).

**Figure 5:**
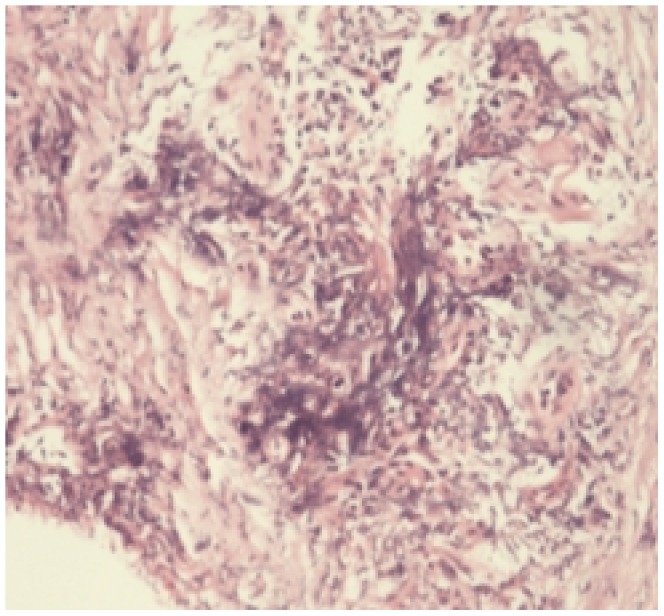
Remarkable neutrophil infiltration. (×100).

**Figure 6:**
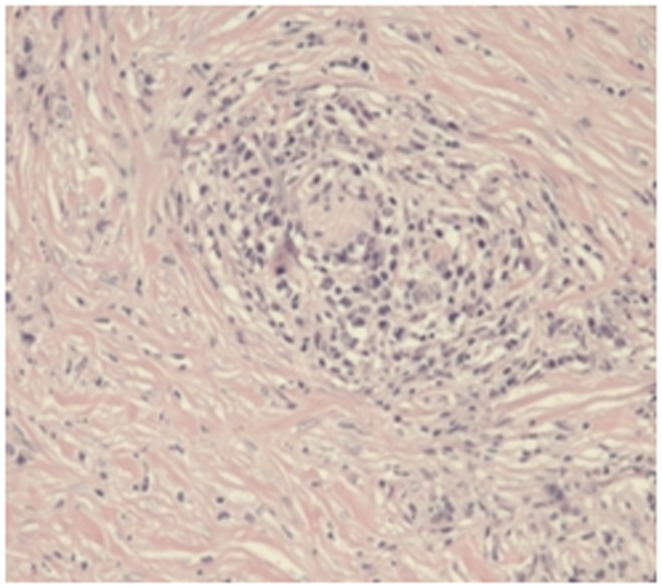
Granulocytic epithelial lesion: lymphoplasmacytic and neutrophilic infiltration with destruction of small duct and ductal epithelium. (×100).

**Figure 7:**
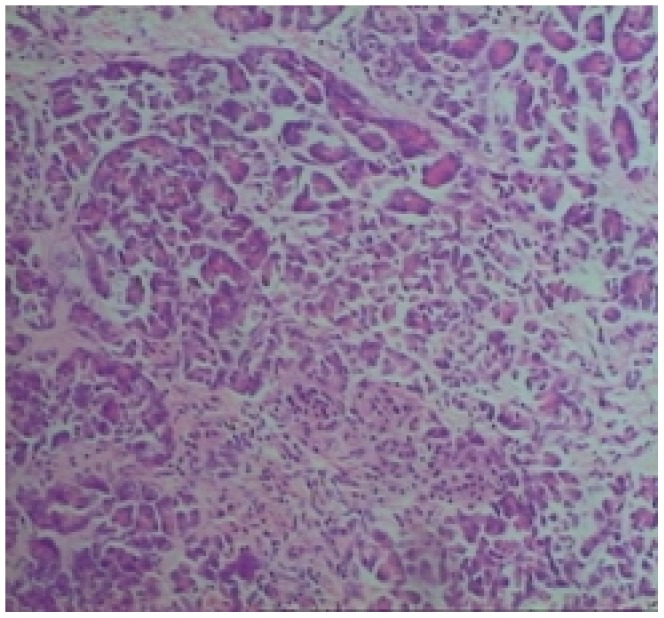
Massive eosinophil infiltration. (×100).

Storiform fibrosis (Figure 1) and infiltration of lymphocytes and plasma cells (Figure 2) were distributed along the main pancreatic duct, as well as along medium- and large-sized interlobular ducts, partially invading small veins within the lesion area, which resulted in irregular stenosis and even obstruction of small veins (obstructive phlebitis) (Figure 3). There were 17 cases (13/29[44.8%] in LPSP; 4/7[57.1%] in IDCP; *P* = 0.684) with partial atrophy of gland alveolus (Figure 4). Seven cases had neutrophil infiltration (Figure 5) with granulocytic epithelial lesion (Figure 6) (4 cases were diffuse and dense; 3 cases were medium to more). Dilated pancreatic duct were seen in 6 cases (4/29[13.8%] in LPSP; 2/7[28.6%] in IDCP; *P* = 0.573). Obliterative phlebitis were found in 5 cases (5/29[17.2%] in LPSP). In addition, we found 3 cases (3/29[10.3%] in LPSP) with nearly complete atrophy of gland alveolus and 2 cases (1/29[3.4%] in LPSP; 1/7[14.3%] in IDCP; *P* = 0.356) with atypical ductal epithelial hyperplasia. Other rare histological manifestations in our patients included 1 case (1/29[3.4%] in LPSP) with fibroblast hyperplasia, 1 case (1/29[3.4%] in LPSP) with interstitial mucinous degeneration, 1 case (1/29[3.4%] in LPSP) with disturbed ductal arrangement, 1 case (1/29[3.4%] in LPSP) with lymph follicle formation, 1 case (1/7[14.3%] in IDCP) with gland hyperplasia, 1 case (1/29[3.4%] in LPSP) with massive eosinophilic infiltration (Figure 7), 1 case (1/29[3.4%] in LPSP) with acidophil substances within the duct, and 1 case (1/7[14.3%] in IDCP) with collagenization. Otherwise, seven cases (7/29[24.1%] in LPSP) had pancreatic calculi, 4 cases (4/29[13.8%] in LPSP) had pancreatic cysts, and 1 case (1/29[3.4%] in LPSP) had both pancreatic a calculus and cyst.

**Figure 8:**
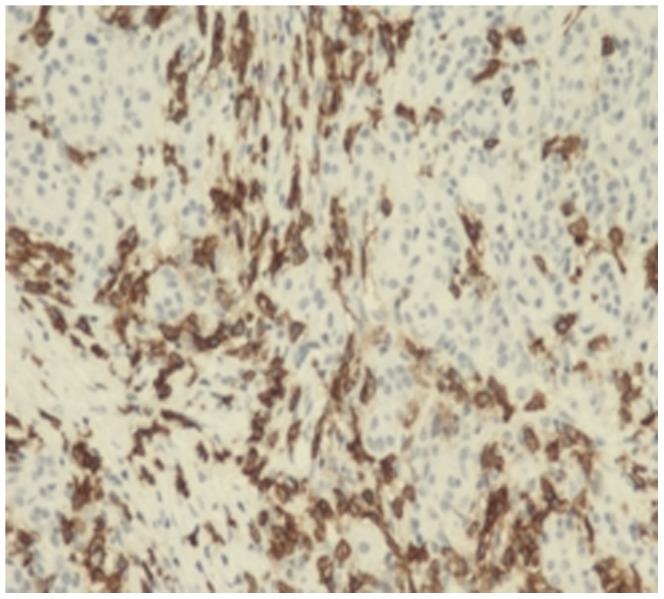
Markedly increased CD38-positive plasma cells infiltration. (×100).

**Figure 9:**
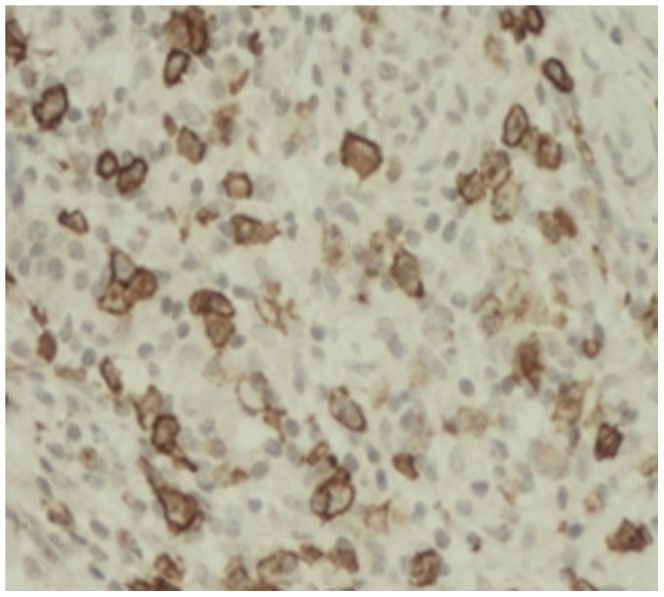
Obviously increased CD138-positive plasma cells infiltration. (×200).

**Figure 10:**
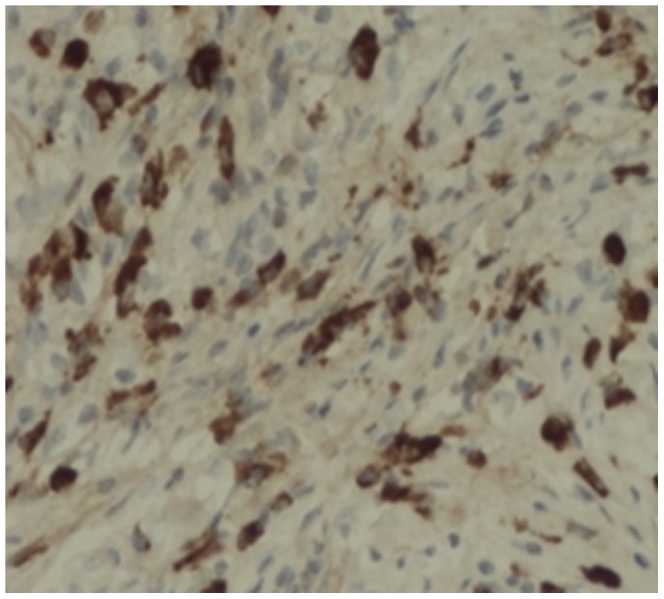
diffuse and dense infiltration by IgG4-positive plasma cells. (×200).

The lymphocytes were CD3(+), CD4(+), CD7(+), CD8(+), CD20(+) and CD79a(+). The plasma cells were CD38(+) (Figure 8), CD138(+) (Figure 9), and mostly expressed IgG4 (Figure 10). The density of IgG4-positive cells varied. Twenty-two (75.9%) cases in LPSP had >50 IgG-positive cells/high-power field (HPF), mean of IgG4-positive cells/HPF were 60 cells/HPF; 5 (17.2%) cases in LPSP had >30 IgG-positive cells/HPF, mean of IgG4-positive cells/HPF were 39 cells/HPF; 2 (6.9%) cases in LPSP had >10 IgG-positive cells/HPF, mean of IgG4-positive cells/HPF were 19 cells/HPF. Seven cases with IDCP did not show any significant staining for IgG4.

### Imageological Data

**Figure 11:**
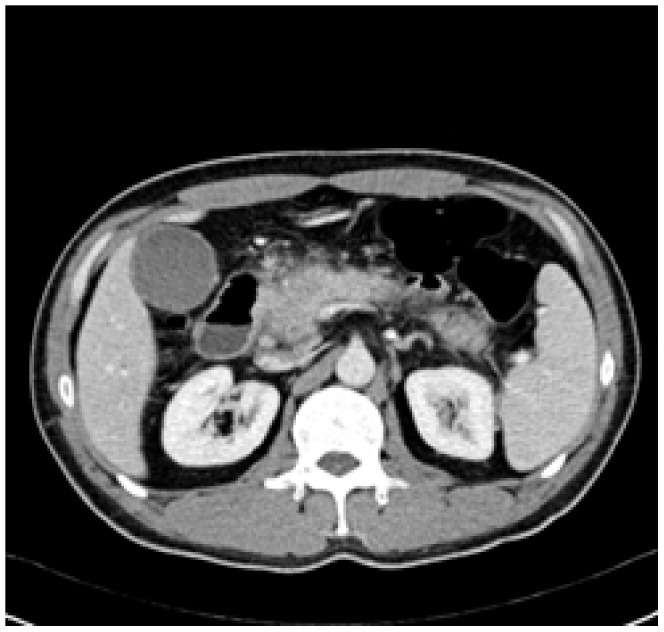
Enhanced CT showing nodules surrounding head of pancreas and interrupted common bile duct at the lesion.

**Figure 12:**
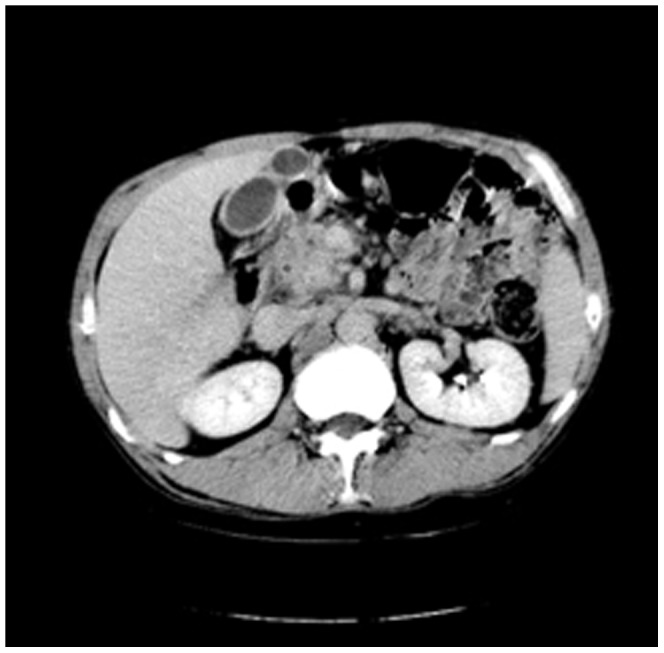
Enhanced CT showing a capsule-like rim.

**Figure 13:**
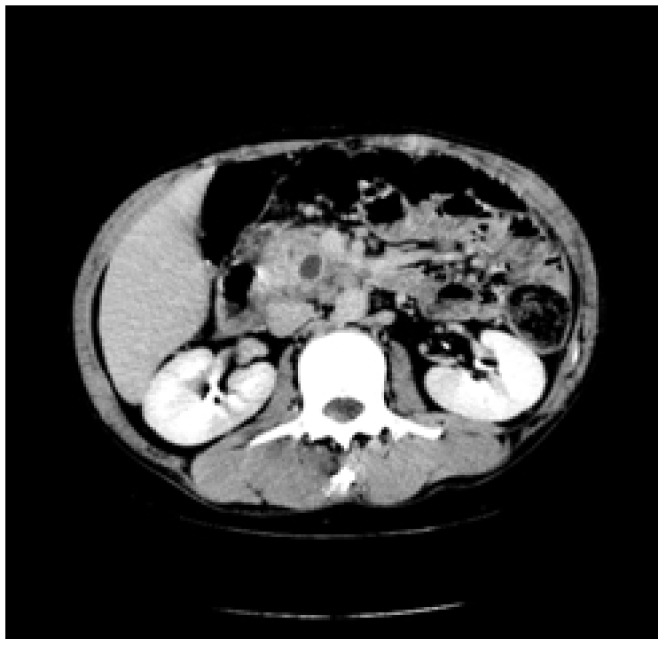
Enhanced CT revealing enlargement and cystic low-density shadow of head of pancreas.

**Figure 14:**
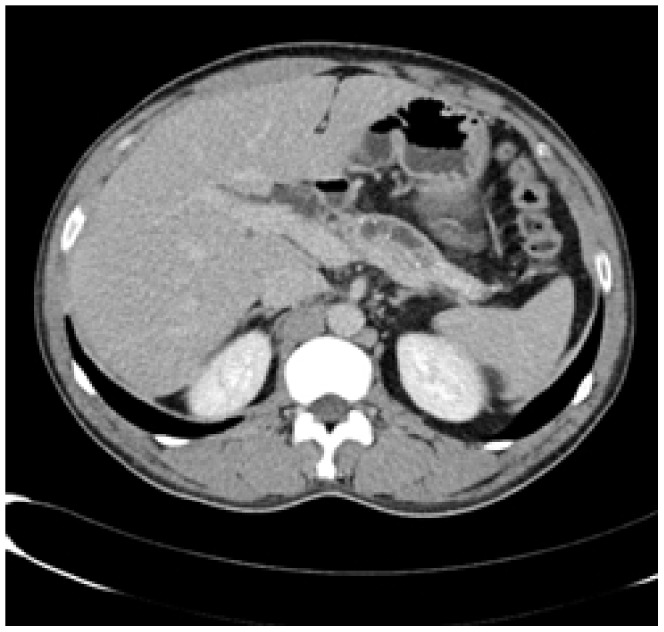
Enhanced CT revealing dilation and string-of-beads change of pancreatic duct,calcification or pancreatic calculus.

**Figure 15:**
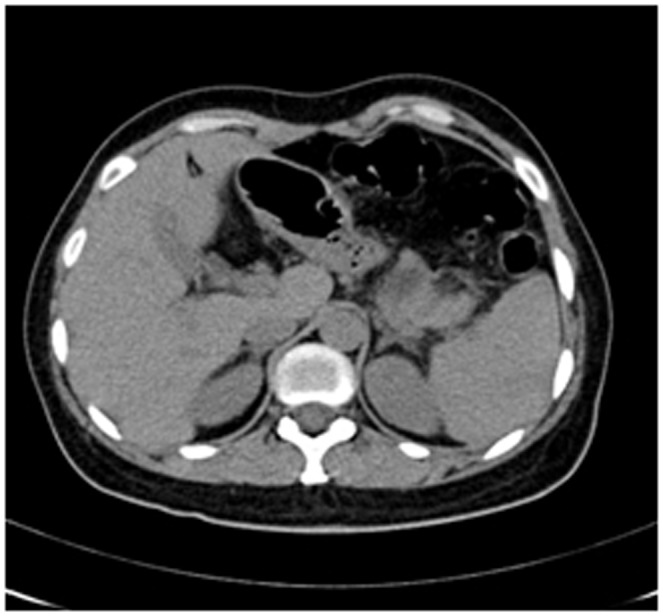
Enhanced CT showing peripancreatic narrow shadows.

Enhanced CT for 27 cases (22 in LPSP; 5 in IDCP): In most cases, the pancreas was “banana shaped” or “sausage shaped” instead of the “feather shaped” normal pancreas. There were 11 cases (8/22[36.4%] in LPSP; 3/5[60%] in IDCP; *P* = 0.37) in which the pancreatic head was enlarged or contained a mass (nodules surrounding the pancreatic head in 1 case and the pancreatic head with lobular architecture in 1 case) (Figure 11), 10 cases (9/22[40.9%] in LPSP; 1/5[20%] in IDCP; *P* = 0.621) with a capsule-like rim (“halo”sign) (Figure 12), 4 cases (4/22[18.2%] in LPSP) with a cystic low-density shadow in the pancreatic head (Figure 13), 4 cases (4/22[18.2%] in LPSP) with a generally enlarged pancreas, 2 cases (1/22[4.5%] in LPSP; 1/5[20%] in IDCP; *P* = 0.342) with enlargement of the body and tail of the pancreas, 2 cases (2/22[9.1%] in LPSP) with an enlarged pancreatic tail (multiple fluid sonolucent areas in 1 case), 1 case (1/22[4.5%] in LPSP) with a cystic mass of the pancreatic tail, 1 case (1/22[4.5%] in LPSP) with pancreatic atrophy. Also, we obtained the data of 7 cases (6/22[27.3%] in LPSP; 1/5[20%] in IDCP; *P* = 1) with calcification or pancreatic calculi (Figure 14), 4 cases (3/22[13.6%] in LPSP; 1/5[20%] in IDCP; *P* = 1) with narrow or patchy exudation in peripancreatic lesions, 3 cases (3/22[13.6%] in LPSP) with a few fibroidlike stripes in peripancreatic lesions (Figure 15), 2 cases (2/22[9.1%] in LPSP) with peripancreatic lymph node enlargement, 2 cases with increased peripancreatic fat density in peripancreatic lesions, 1 case (1/22[4.5%] in LPSP) with an occupying lesion of the duodenal papilla. The images in 20 cases were delayed enhancement without specific enhanced features. Surprisingly, 1 case (1/22[4.5%] in LPSP) had normal pancreatic shape and density. Thirteen cases (10/22[45.5%] in LPSP; 3/5[60%] in IDCP; *P* = 0.648) revealed dilation of pancreatic duct (full-length string-of-beads dilation in 1 case and interrupted pancreatic duct at the lesion in 1 case) (Figure 14). Furthermore, 8 cases (5/22[22.7%] in LPSP; 3/5[60%] in IDCP; *P* = 0.136) found choledochectasia, 3 cases (3/22[13.6%] in LPSP) showed stenosis of the lower common bile duct and 2 cases (2/5[40%] in IDCP) had an interrupted common bile duct at the lesion (Figure 11).

Enhanced MR imaging (MRI) was performed in 2 cases. In 1 case of IDCP, the pancreatic body and tail were enlarged (lower T1 and slightly higher T2), with uneven enhancement. In the other case of LPSP, the pancreatic head was enlarged in lobular architecture (identical T1 and T2), with obvious enhancement, as well as exudation surrounding the pancreatic head and mild dilation of the main pancreatic duct.

**Figure 16:**
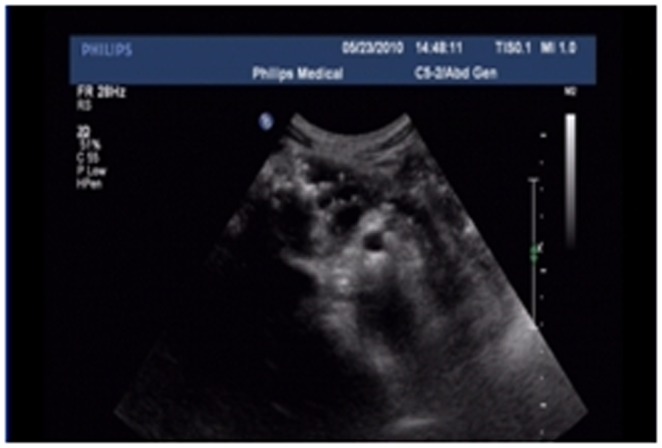
Color Doppler ultrasonography showing string-of-beads dilation of pancreatic duct.

Color Doppler ultrasonography was performed in 18 cases (14 in LPSP; 4 in IDCP). The echo was generally coarse and at a low level. Findings included a mass or enlargement of the head of the pancreas in 12 cases (9/14[64.2%] in LPSP; 3/4[75%] in IDCP; *P* = 1) (cystic for 1 case), generalized enlargement of pancreas in 4 cases (3/14[21.4%] in LPSP; 1/4[25%] in IDCP; *P* = 1) (fluid sonolucent area in the tail of pancreas for 1 case), mass of the pancreatic tail in 3 cases (3/14[21.4%] in LPSP [parenchymal in 2 cases and cystic in 1 case]). Calcification and pancreatic calculi were seen in 2 cases. It happens that there were two similar cases (1/14[7.1%] in LPSP; 1/4[25%] in IDCP; *P* = 0.405) in pancreatic normal shape comparing with the former one case with enhanced CT.One case showed choledochectasia (1/14[7.1%] in LPSP). In 12 cases (8/14[57.1%] in LPSP; 4/4[100%] in IDCP; *P* = 0.245), the pancreatic duct was dilated (average, 0.421 cm with all AIP in diameter; range, 0.19–1.1 cm; average, 0.4625 cm in LPSP and 0.3375 cm in IDCP; *P* = 0.474), and among them there were 10 cases <0.6 cm in diameter (6/12[50%] in LPSP; 4/4[100%] in IDCP; *P* = 0.515) (string-of-beads dilation for 1 case) (Figure 16).

Electronic endoscopy was performed in 5 cases (4 in LPSP; 1 in IDCP): The echo was generally coarse and at a low level. There were 2 cases (2/4[50%] in LPSP) with an uneven image (pseudo lobular change for 1 case), 2 cases (2/4[50%] in LPSP) with a mass in the pancreatic head, 2 cases (1/4[25%] in LPSP; 1/1[100%] in IDCP; *P* = 0.4) with a dilated pancreatic duct and 3 cases (3/4[75%] in LPSP) with calcification or pancreatic calculus.

Endoscopic retrograde cholangiopancreatography (ERCP) was performed in 2 cases of IDCP. One had stenosis of the pancreatic segment of the common bile duct and the other had sudden interruption at the pancreatic segment of the common bile duct.

**Figure 17:**
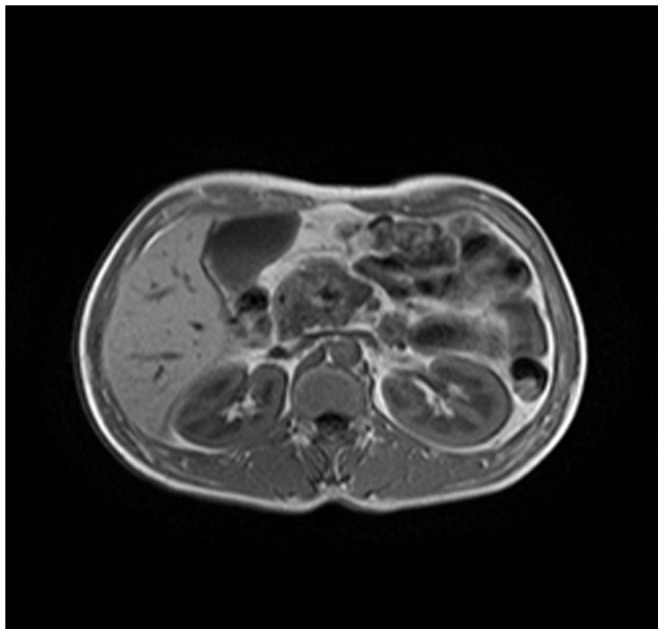
MRCP (T1WI) showing a capsule-like rim.

**Figure 18:**
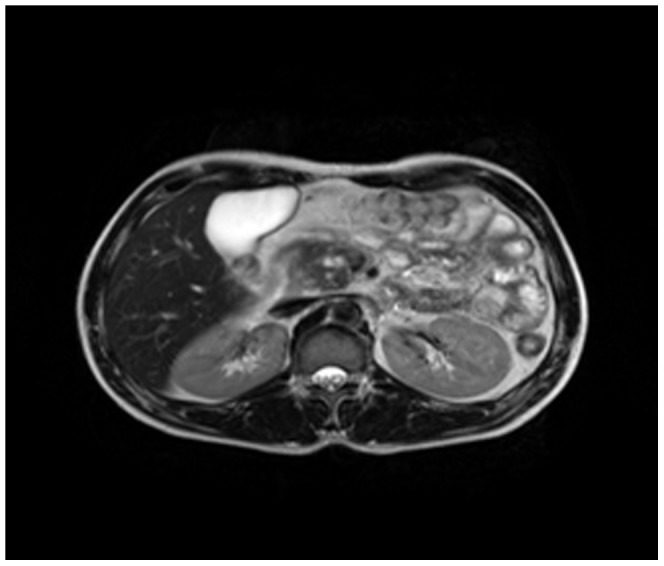
MRCP (T2WI) revealing a capsule-like rim.

**Figure 19:**
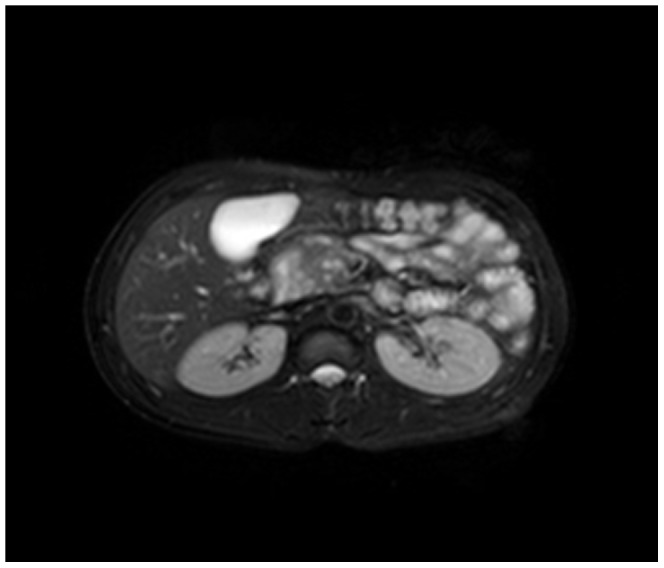
MRCP (T2WI-STIR) revealing a capsule-like rim.

**Figure 20:**
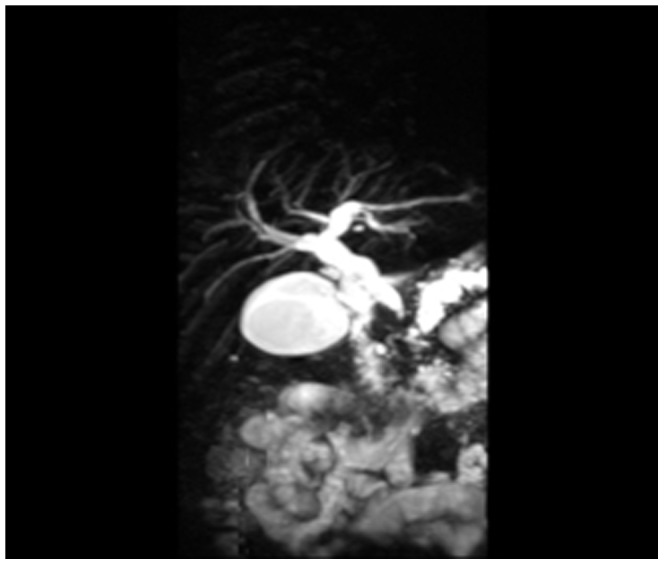
MRCP showing dilation and interruption of pancreatic duct,beak-shaped stenosis of lower common bile duct.

**Figure 21:**
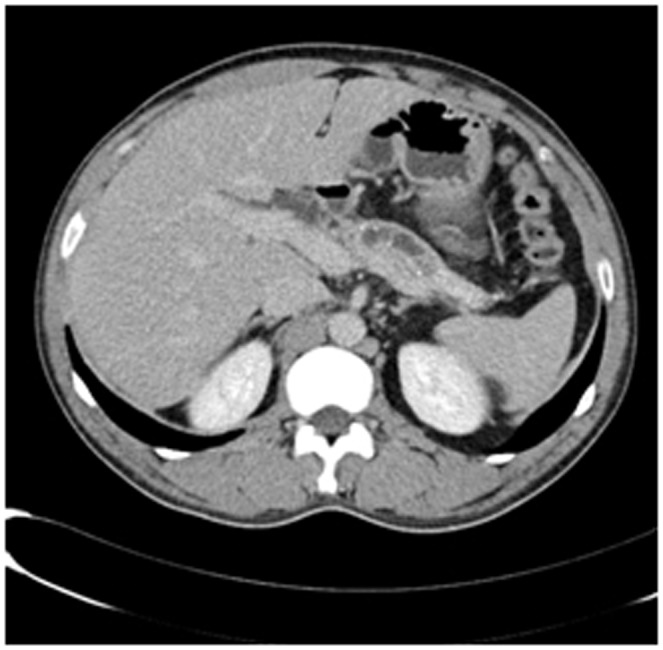
MRCP showing dilation of pancreatic duct string-of-beads dilation of body and tail of pancreas.

Magnetic resonance cholangiopancreatography (MRCP with 3.0 T) was performed in 13 cases (10 in LPSP; 3 in IDCP). The pancreas was uneven in density and irregular in shape. With MRCP we found 10 cases (9/10[90%] in LPSP; 1/3[33.3%] in IDCP; *P* = 0.108) with a capsule-like rim (slightly lower T1 and lower T2) (Figure 17, 18, 19), 8 cases (6/10[60%] in LPSP; 2/3[66.7%] in IDCP; *P = 1*) with an enlarged pancreatic head (lower T1 and identical T2 or slightly longer T2), 5 cases (4/10[40%] in LPSP; 1/3[33.3%] in IDCP; *P* = 1) with atrophy in the pancreatic body and tail lesions (longer T1 and T2), 2 cases (1/10[10%] in LPSP; 1/3[33.3%] in IDCP; *P* = 0.423) with a cystic lesion occupying the head of the pancreas (longer T1 and T2), 2 cases (1/10[10%] in LPSP; 1/3[33.3%] in IDCP; *P* = 0.423) with a distended pancreas (lower T1 and slightly longer T2), 1 case (1/10[10%] in LPSP) with an enlarged pancreatic tail (longer T1 and T2), 1 case (1/3[33.3%] in IDCP) with lymph node enlargement in peripancreatic lesions and 1 case (1/10[10%] in LPSP) with normal pancreatic shape. The 9 cases (8/10[80%] in LPSP; 1/3[33.3%] in IDCP; *P* = 0.203) with a dilated pancreatic duct (2 cases with irregular dilation; 1 case with cystic dilation; 1 case with full-length dilation; 1 case with string-of-beads dilation; and 1 case with interruption at the pancreatic head) (Figure 20, 21). MRCP found 9 cases (8/10[80%] in LPSP; 1/3[33.3%] in IDCP; *P* = 0.203) with beak-shaped stenosis at the lower common bile duct (Figure 18, 19, 20) and 7 cases (6/10[60%] in LPSP; 1/3[33.3%] in IDCP; *P* = 0.559) with choledochectasia.

**Figure 22:**
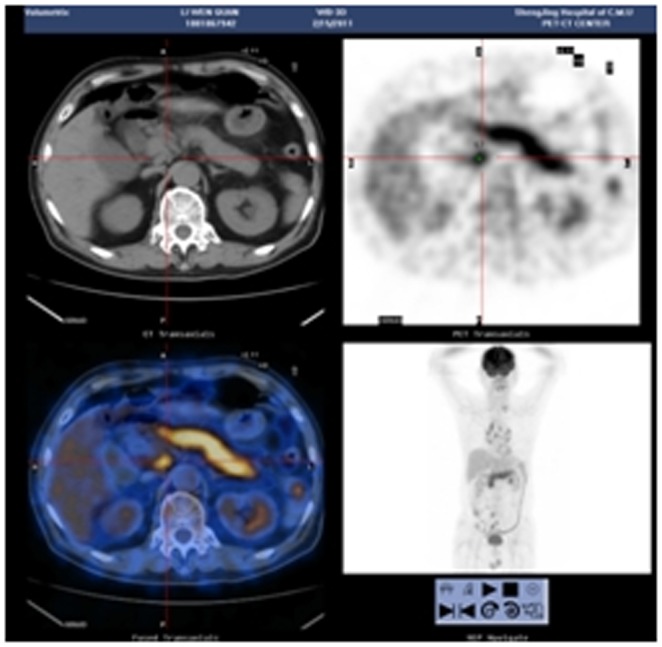
PET-CT revealing swelling and pencil sharp of pancreas with enlargement of peripancreatic lymph nodes.

Positron emission tomography-computed tomography (PET-CT) was performed in 1 case of LPSP. The whole pancreas was swelling and pencil sharp in architecture with an uneven density in parenchyma, and the lymph nodes in peripancreatic lesions were slightly enlarged (Figure 22).

## Discussion

AIP as the benign disease resembles pancreatic carcinoma both clinically and radiographically. Kamisawa et al [5] believed that AIP was not only a type of pancreatitis but also a kind of pancreatic injury resulting from systematic diseases. This injury was primarily manifested as AIP, sclerosing cholangitis, sclerosing sialadenitis (including Mikulicz’s disease and Kuttner’s tumor), and retroperitoneal fibrosis, accompanied with lymphadenopathy, as well as occasional inflammatory pseudotumor which was easily misdiagnosed as pancreatic cancer, which is therefore treated surgically. Horiuchi et al [6] classified AIP into diffuse type and focal types; the former developed from the later [7]. The focal type was a variant type of AIP [8] and relatively rare, most frequently invading the head of pancreas. Diffuse swelling of the pancreas likely reflected an earlier phase in the evolution of pancreatic inflammation in AIP [9]. Recently it has been suggested that the term “AIP” encompasses 2 different subtypes of the disease with distinct histopathology and clinical profile [10]. The histologic pattern in type 1 was called LPSP, which was characterized by periductal lymphoplasmacytic infiltration, storiform fibrosis, and obliterative venulitis [11]–[12]. The histologic pattern in type 2 was called IDCP, the hallmark of which were neutrophil infiltration and the granulocytic epithelial lesions [13]. Far less was known about type 2 than type 1.

Several reports had revealed that pathological classifications were associated with clinical features, serological test, and imageological profiles. It was definite in our cohort that most cases (80.6%, 29/36) of AIP fit the concept of LPSP. All 36 cases in this report were all misdiagnosed preoperatively as pancreatic cancer, ordinary pancreatitis, or duodenal cancer, revealing that AIP was usually misdiagnosed. The aim of the retrospective analysis performed in this study was to improve the identification and diagnosis strategies for AIP in China.

In 2002, Japanese researcher Nishimori et al [14] found in epidemiologic survey that the morbidity of AIP was 0.82/100,000 persons in Japan, the age of onset was frequently over 45 years and the male/female ratio was near 3∶1. AIP was more prevalent in males (approximately 2∶1) and usually occured later in adulthood, with the majority of patients over 50 years in age [15]. AIP may be seen in any age group, it has been reported in a 10 year-old child [16]. In this study, 75% (27/36) of the patients were ≥45 years and there was a male/female ratio of 4.1∶1, similar to the demographics described in Japan. The type 1 in this series showed a sex predilection (the ratio of male/female was 6.25/1). Patients with the type 2 were, on average, a decade younger than the type 1 patients and didnot showed a gender predilection. AIP has no specific symptoms. The biliary tree was the most common site of extrapancreatic involvement, characterized by bile duct wall thickening, stenosis, and stricturing [17]. It was reported that about 50% of patients visited a doctor due to yellow skin discoloration [18]. Severe abdominal pain was rare. The most frequent acute presentation of AIP was with obstructive jaundice and/or pancreatic mass [3]. To date, no critical necrotizing pancreatitis has been reported [19]. Obstructive jaundice occured in 76% and weight loss in 35% of patients [20]. Another reported that obstructive jaundice was frequent in AIP patients, occurring in one-third of patients [21]–[22]. This study found that the most common clinical manifestations of AIP were body weight loss (52.78%), upper abdominal pain (50%) and jaundice (41.67%). Patients with type 1 (51.7%) were close to those with type 2 (57.1%) in weight loss, patients with type 1 (55.2%) were more likely with upper abdominal pain than those with type 2 (28.6%), patients with type 1 (37.9%) were less likely with jaundice than those with type 2 (57.1%), but differences of three symptoms between type 1 and type 2 were not statistically significant. The jaundice was transient. Weight loss was probably the results of impaired digestion and decreased appetite. The most frequent complications were chronic cholecystitis (47.22%), diabetes mellitus (16.67%) and RBBB (16.67%). It was uncertain that the cardiac change was the contributing factor of heart involvement by AIP. No heart conduction block was yet reported. Patients with type 1 (41.4%) were less likely with chronic cholecystitis than those with type 2 (71.4%), patients with type 1 (20.7%) were more likely with diabetes mellitus than those with type 2 (0%), patients with type 1 (20.7%) were more likely with RBBB than those with type 2 (0%), but differences of three complications between type 1 and type 2 were not statistically significant. The Japan experts reported that diabetes mellitus was often (43%–68%) observed in patients with AIP [23]–[24]. Diabetes mellitus was frequent in our patients with type 1, a result that might be explained by the older age. It was reported that stomach [5], [25]–[27], papilla of Vater [28], intestine [27]–[29], kidney [30]–[31], lymph node [32], lung [33] and thyroid gland [34] might be involved in patients with AIP. Because the obtained data in our patients were not directly supportive, it was unclear that whether the changes of other organs observed in our patients were due to AIP such as hepatic cirrhosis, pleural effusion, peritoneal effusion, duodenal papillitis, myocardial ischemia and renal insufficiency. Even the initial symptom for two patients was abnormal BIL occasionally found in the physical examination. The involvement of salivary gland, lacrimal gland and endocrine gland could result in painless gland enlargement, dry eyes and mouth, Hashimotos thyroiditis, hypophysitis [35], etc. The invasion of central nervous system was extremely rare, and there were only a few reports of ependymal inflammatory pseudotumor [36] and pachymeningitis [37].

Based on Kamisawa T et al [38] reports, the clinical features for young patients (<40 years) were different from those of middle-aged and elderly patients (≥40 years); abdominal pain and increased blood diastase were more common in the young patients. The reported percentage of young patients with abdominal pain as chief complaint (100%) was significantly higher than that of middle-aged and elderly patients (43%). Fewer young patients (17%) had obstructive jaundice compared with middle-aged and elderly patients (59%); in contrast, blood diastase was more frequently increased in young patients (83%) than in middle-aged and elderly patients (40%). The corresponding values in this study were 87.5% vs. 42.9%, 37.5% vs. 57.1% and 42.9% vs. 46.1%, respectively, showing the trends that were similar to the previous reports. Thus, we believe that the manifestations of AIP in the young group are similar to acute, ordinary, pancreatitis, with acute onset; while AIP in the older group is characterized by insidious onset.

Our laboratory findings showed that AIP could often involve the hepatic and biliary systems. The serum hepatic and biliary enzymes as well as BIL increased in many AIP patients, especially with a markedly increased γ-GT (79.4%) level. Patients with type 1 (67.9 umol/L; 48.9 umol/L; 17.5 umol/L) were close to those with type 2 (60.7 umol/L; 38.8 umol/L; 22 umol/L) in TBIL, DBIL and IBIL. All had no statistical significance. However, patients with type 1 (160.1 U/L; 267.4 U/L; 503.7 U/L) were inferior to those with type 2 (332.1 U/L; 593.4 U/L; 1043.7 U/L) in ALT, ALP and γ-GT with statistical significance (*P* = 0.044, 0.025 and 0.013), respectively. Patients with type 1 (102.2 U/L) were inferior to those with type 2 (165.9 U/L) in AST with difference close to statistical significance (*P* = 0.072). There may be an elevated CA19-9[39]–[40], and CA19-9 was elevated in 53% of cases, probably because of cholestasis [6], [41]–[42]. CA19-9 in this study was elevated in 35% of cases, and the values in IDCP were higher than in LPSP. Notably, the remarkably increased CA19-9 cannot be used as the basis to exclude AIP. Our results indicated that the type 2 patients had significantly increased diastase (inflammatory indicator) while most type 1 patients suffered obstructive jaundice (sclerosis-caused compression) which was consistent with Nishimori I et al studies. Lü H et al [43] reported 16 AIP patients, 31.2% of whom had increased blood lipase and 18.7% had a transient increase in blood diastase. Law et al [44] reported that serum amylase and lipase were neither sensitive nor specific for AIP. In this study, 7 of 8 (87.5%) AIP patients had an increased blood lipase, and 9 of 20 (45%) had transiently increased blood diastase, which were both higher than the results reported by Lü H. After careful analysis, we found that the increases of lipase and diastase in LPSP were higher than in IDCP without statistical significance. This may be due to the smaller sample size for the blood lipase test, and needs to be confirmed by using a large sample size.

In addition, approximately 20%–40% of AIP patients have other autoimmune diseases, such as systemic lupus erythematosus, Sjogren syndrome, Crohn’s disease, primary biliary cirrhosis and primary sclerosing cholangitis, etc [32], [45]–[48].

Some AIP patients have various autoantibodies present, including ANA, AMA, antismooth muscle antibody, antimicrosomal antibody, antineutrophil cytoplasmic antibody(especially p-ANCA), antithyroglobulin antibody and rheumatoid factor. However, these antibodies were found to be transient, and even when levels of IgG and IgG4 were normal, autoantibodies were still present in nearly 5% of patients. This suggests the presence of IgG4 and autoantibodies is only supportive, but not completely specific, to the diagnosis of AIP. As for one finding in this study that anti-Ro-52 antibody was positive, to date there are no relevant reports. A serum IgG4 of >1350 mg/L has high sensitivity and specificity in the diagnosis of AIP [7], and a serum IgG4≥2200 mg/L for patients diagnosed as AIP often indicates not only pancreatic injury but also extrapancreatic organ involvement [49]. However, increased IgG4 cannot support a definite diagnosis of AIP, because a few patients with pancreatic cancer also have a significantly increased IgG4. In addition, IgG4 may increase in patients with pemphigus, bronchial asthma or atopic dermatitis [50]. Because the clinical measurement of serum IgG4 levels was not introduced in the period of selected cases in hospital, it was impossible to provide the detailed information on serum IgG4 levels. The measurement of immunological indice (such as IgG and antinuclear antibody series) were performed on only a few patients for insufficient understanding of AIP before. With more understanding of AIP, at present we are strengthening the measurement of immunological indice for AIP. We have recently been allowed to develop clinical serum IgG 4 level detection.

The typical imageological finding in AIP is diffuse pancreatic enlargement in the shape of a banana or sausage. This study most frequently found a mass or enlargement in the pancreatic head, whether assessed by the enhanced CT (11/27[40.7%]), byenhanced MRI (1/2[50%]), by color Doppler ultrasonography (12/18[66.7%]), by electronic endoscopy (2/5[40%]), or by MRCP (8/13[61.5%]). The enhancement signals by the enhanced CT were non-specific. A well-defined capsule-like rim (“halo” sign) which can be seen in 12%–40% of patients with AIP [34], [39]–[40], [51]–[52] was common by using enhanced CT (10/27[37%]) and MRCP (10/13[76.9%]), and was caused by fibrosis surrounding the lesions. This “halo” sign is an important imageological characteristic of AIP and is rare in malignant pancreatic tumors or ordinary CP [15]. The enhancement delay that was common with enhanced CT (20/27[74.1%]) might be associated with the lymphoplasmacytic infiltration and fibrosis [53]. CT shows a dilated main pancreatic duct in some occasions [38]. Thirteen cases (13/27[48.1%]) evaluated by enhanced CT in this study were found to have dilation of the pancreatic duct. As reported, the extent of dilation of distal pancreatic duct was mild or moderate compared with pancreatic cancer, usually no more than 0.6 cm in diameter [5]. The ductal compliance was confirmed indirectly by color Doppler ultrasonography in our study (i.e., the pancreatic duct diameter in 10 cases [83.3%] did not exceed 0.6 cm) although pancreatic duct diameter was not measured by CT. Calcification or pancreatic calculus was observed in 7 cases with enhanced CT (7/27[25.9%]), in 2 cases with color Doppler ultrasonography (2/18[11.1%]) and in 3 cases with electronic endoscopy (3/5[60%]). Furthermore, cystic degeneration was seen in 5 cases by using enhanced CT (5/27[18.5%]), in 3 cases by using color Doppler ultrasonography (3/18[16.6%]) and in 2 cases by using MRCP (2/13[15.4%]). It was inferred that imageological changes of these cases were due to marked inflammation. Exudation, stripes and lymph node enlargement in peripancreatic lesions were relatively rare, thus mild inflammatory reaction should be considered [51]. As the other organ involvement in AIP can precede pancreatic involvement [54], normal pancreatic shape and density was not an exclusion criterion for AIP, and rarely, no abnormality may be seen on cross-sectional imaging [3]. An enhanced MRI showed a pancreatic head with lobular architecture and the enhancement signals were nonspecific. Because the MRCP can show diffuse thinning of main pancreatic duct [55], non-visualization of the main pancreatic duct on MRCP might suggest narrowing of the main pancreatic duct. Electronic endoscopy may be a useful adjunct to differentiate concentric bile duct thickening (more commonly seen in AIP) from strictures caused by extrinsic compression as seen in pancreatic cancer [56]–[57]. Kamisawa et al [58] believed that MRCP (1.5 T) could not be used as a substitute for ERCP in the diagnosis of AIP but might be employed for follow-up. There is also a wide variation in clinical practice across the globe. For example, ERCP is routinely used for investigating obstructive jaundice in Japan and is a mandatory criterion in the Japanese criteria [59]–[60], but MRCP is also available in Korean. MRCP has become popular as a non-invasive method for obtaining high quality images of the pancreaticobiliary tree [61]. Endoscopists in the western countries generally avoid injecting the pancreatic duct in patients with obstructive jaundice for fear of causing pancreatitis, and AIP in the West is diagnosed without a requirement for ERCP. According to our long-term clinical experience, noninvasive examinations are easily accepted by patients in China. It is a risk factor to increase the rates of infectious diseases and acute pancreatitis by using ERCP and electronic endoscopy, especially in acute phase. Otherwise, MRCP in our hospital is done by using a 3.0T MRI machine which is obviously superior to 1.5T in image resolution. Hence, it is practical for patients in China to select MRCP instead of ERCP. Our findings revealed that the enhanced CT, color Doppler ultrasonography, ERCP and MRCP all showed string-of-beads dilation of the pancreatic duct, which was a distinctive change in AIP. In this study, the PET-CT showed that the whole pancreas was swelling and pencil sharp in architecture as a result of inflammation and fibrosis at the affected site. As reported, because these two diseases had different structural forms, the PET-CT was helpful for distinguishing AIP from pancreatic cancer. Pancreatic cancer was in nodular architecture while AIP was in smoother ‘sausage-like’ architecture. Furthermore, it was believed in this study that the PET-CT might facilitate the detection of multiple systematic lesions. Moreover, MRCP images of AIP showed beak-shaped stenosis at the lower common bile duct and interruption of common bile duct and pancreatic duct at the lesion, which were easily confused with malignant diseases.

Histologic evaluation remains the gold standard for diagnosis of AIP. The histologic findings in our patients included lymphoplasmacytic infiltration with or without neutrophil, storiform fibrosis and obliterative phlebitis, which were essential to establishing the diagnosis of AIP. There was often associated with infiltration of eosinophils and fibroblasts. LPSP and IDCP share some histopathological features, such as periductal lymphoplasmacytic infiltration and storiform fibrosis. Lymphoplasmacytic infiltration and fibrosis destroy the vein wall and disrupt its elastin fibers, resulting in narrowing and even occlusion. If specific features such as GELs or obliterative phlebitis were identified, they were almost pathognomonic for AIP, with GELs favoring type 2 and obliterative phlebitis favoring type 1 [13]. In severe cases, the fibrotic changes can encompass large areas, with myofibroblasts arranged in a storiform pattern resembling an inflammatory pseudotumor [62].

The pathological investigation indicated in our patients that changes in the head of the pancreas were most frequent in AIP, i.e., 25%. The changes of pancreatic head were more frequently seen in type 2 than in type 1 (*P* = 0.05). IgG4-positive plasma cell infiltration is widely accepted as the gold standard for the diagnosis of AIP, as observed in most specimens in this study. Moderately (10–30/HPF) increased IgG4-positive plasma cell infiltrates are seen in 72% to 100% of AIP [13]. Immunolabeling for IgG4 demonstrated that positive staining of plasma cells with the density of positive cells were correlated with the subtypes of AIP. We observed that the combination of higher (>30/HPF) and dense (>50/HPF) IgG4 labeling of plasma cells was up to 93.1% (27/29) in the type 1 cases. Our data suggested that within most affected areas, the IgG4 distribution was often diffuse and type 1 might be etiologically different from type 2. The European study used more than 20 positive cells/HPF as a cutoff. Higher and dense immunoexpression of IgG4 in plasma cells at a density of over 30 or more cells per HPF appeared to be a specific finding in China with type 1. However, Kojima et al [63] reported that positive IgG4 cells were seen in 72.5% of AIP patients and in 63.1% of non-AIP patients with CP, suggesting that IgG4-positive plasma cell infiltration cannot be used as the unique basis for the diagnosis of AIP and that serological and imageological characteristics must be considered. Atropy or calcification was the atypical presentation in Mayo Clinic Criteria. This study found that complete or partial gland alveolus atrophy was most frequently seen, cystic degeneration and pancreatic calculus were less frequently observed, dilation of pancreatic duct and fatty nodules surrounding the lesions were found. Lymph follicle formation, collagenization and interstitial mucinous degeneration were all rare in our patients. Seven cases in this study were found to have pancreatic calculus by pathological examination. Kamisawa and Okamoto [64] found 55% recurrent patients developed pancreatic calculus in different periods of time. Kawa et al [65] described 42 patients who finished a 12-month follow-up, of whom 8 cases and half of the recurrent cases developed pancreatic calculus. Hence, AIP is considered as a progressive disease that eventually leads to the development of pancreatic calculus, similar to other types of CP. To be concluded from our pathological data, it is clear that destruction of the pancreatic parenchyma architecture is due to obvious lymphoplasmacytic infiltration, the inflammatory response and fibrosis disrupting the venous endothelium that often results in obliterative phlebitis. Fibrosis may also affect the acinar tissue and produce profound lobular atrophy. In China, plasma cells staining of IgG4 at a density of over 30 or more cells per HPF appear to be a specific finding with type 1, and granulocytic epithelial lesions with plasma cells staining of IgG4-negative are pathognomonic for type 2.

The limitation of our study is that all of the patients in the series were identified retrospectively as AIP was not a well-recognized entity in China before. Recently, the clinical measurement of IgG4 has been introduced in our hospital. It is believed that as we become more familiar with the features of AIP, more patients in China will be diagnosed with AIP and treated, avoiding unnecessary surgery.

In summary, AIP patients were usually middle-aged or elderly, and had an acute or insidious onset, often associated with body weight loss. Type 1 showing a sex predilection (males) was commonly more dominant than type 2 in all AIP. Type 2 without a gender predilection was, on average, a decade younger than type 1. Their most frequent clinical manifestations were nonspecific and included abdominal pain, abdominal distension, jaundice often complicated by biliary system disorders, possible diabetes mellitus and cardiac conduction block (especially right bundle branch). They had elevated blood levels of hepatic, biliary and pancreatic enzymes as well as tumor marker CA19-9. Type 1 was inferior to type 2 in ALT, ALP and γ-GT with statistical significance (*P = *0.044, 0.025 and 0.013). Type 1 was inferior to type 2 in AST with difference close to statistical significance (*P = *0.072). Histopathology revealed frequent lymphoplasmacytic infiltration with less frequent infiltration of neutrophils, eosinophils and fibroblasts. Diffuse and intensive interstitial fibrosis could be seen. Gland alveolus atrophy was observed to a variable extent. Cystic degeneration, pancreatic calculi, dilation of pancreatic duct and fatty nodules surrounding the lesions were visible. But lymph follicle formation, collagenization, and interstitial mucinous degeneration were rarely revealed. The changes of pancreatic head were more frequently seen in type 2 than in type 1 (*P* = 0.05). Plasma cells staining of IgG4 at a density of over 30 or more cells per high-power field appeared to be a specific finding in China with type 1. Imageology found a diffusely or focally enlarged pancreas, most frequently a mass or enlargement in the pancreatic head, characteristic capsule-like rim, calcification or pancreatic calculus and cystic degeneration. However, we rarely found the images of exudation, stripes and enlarged lymph nodes in peripancreatic lesion. Pancreatic atrophy, normal pancreatic in shape and density, and string-of-beads dilation of pancreatic duct were scarcely seen on radiology. Imageology frequently showed stenosis of the common bile duct.

AIP is a newly recognized chronic disease that has no typical symptoms and signs, that is very easily misdiagnosed as pancreatic cancer and consequently subject to unnecessary surgical treatment. It is also frequently misdiagnosed as ordinary CP, which delays the best treatment with steroids and immunosuppressants. Thus, routine of serum assays should be performed for pancreatic mass, hepatic dysfunction, or hypergammaglobulinemia. The diagnosis of AIP will be greatly improved by judiciously using pancreatic biopsy and testing with new markers. The primary features of AIP in the diagnostic criteria proposed by different groups are derived from their own experience. Most of the current Asian AIP data are from Japan and Korea. However, in the Chinese population there are rare reports on the onset, clinical characteristics and prognosis of AIP with a large sample size. To better diagnose AIP in Chinese clinical practices, we must enhance the recognition of AIP, improve the diagnostic accuracy of serology, pathology and imageology, and recognize the diagnostic value of the response to a trial of glucocorticoids. Yet the pathogenesis of AIP remains poorly understood. Further studies are needed to clarify the the pathogenetic mechanisms responsible for type 1 and type 2 of AIP.

## Methods

Subjects: All pancreatic specimens whose postoperative diagnoses were nonmalignant diseases at Shengjing Hospital of China Medical University from January 2003 to October 2011 were reviewed. Those showing moderate to severe chronic fibrosing pancreatitis with a predominant lymphoplasmacytic infiltration accompanying (or not) neutrophils were identified, then histologically evaluated with IgG4 staining. A total of 36 patients were included as AIP in the study. Histological confirmations on basis of the Honolulu Consensus Document [66] classified in lymphoplasmacytic sclerosing pancreatitis (LPSP) or AIP without granulocytic epithelial lesions (GELs) and idiopathic duct-centric pancreatitis (IDCP) or AIP with GELs were available in 29 patients (80.6%) and in 7 patients (19.4%), respectively. Preoperative diagnoses in admitted patients were 18 cases with pancreatic cancer, 10 cases with pancreatitis, 3 cases with occupying lesion of the lower common bile duct, 3 with obstructive jaundice, 1 with pancreatic pseudocyst, and 1 with duodenal cancer.

Methods: The clinical phenotypes associated with the histopathologic patterns of LPSP and IDCP were referred to as type 1 and type 2 of AIP, respectively [66]. A retrospective analysis of the clinical features, serological data, pathological findings and imageological records was performed in line with the subtypes of AIP. All procedures in this study were approved by the Ethics Committee of Shengjing Hospital of China Medical University (permission code 2011112) (Figure S1). All patients gave written informed consent.

### Statistics Analysis

Comparisons of the profiles between the two subtype patients were performed by using the Student’s t-test or Mann-Whitney U test for continuous variables and Fisher’s exact test for categorical variables. All analyses were performed with SPSS13.0. *P*<0.05 was accepted as indicating statistical significance.

## Supporting Information

Figure S1 **Ethics committee certification.**
(JPG)
