# Supplementary figures and images for: Clinical Analysis of 36 Cases of Autoimmune Pancreatitis in China
Source: PLoS One. 2012 Sep 18;7(9):e44808. doi: 10.1371/journal.pone.0044808 (PMC3445578; doi:10.1371/journal.pone.0044808)

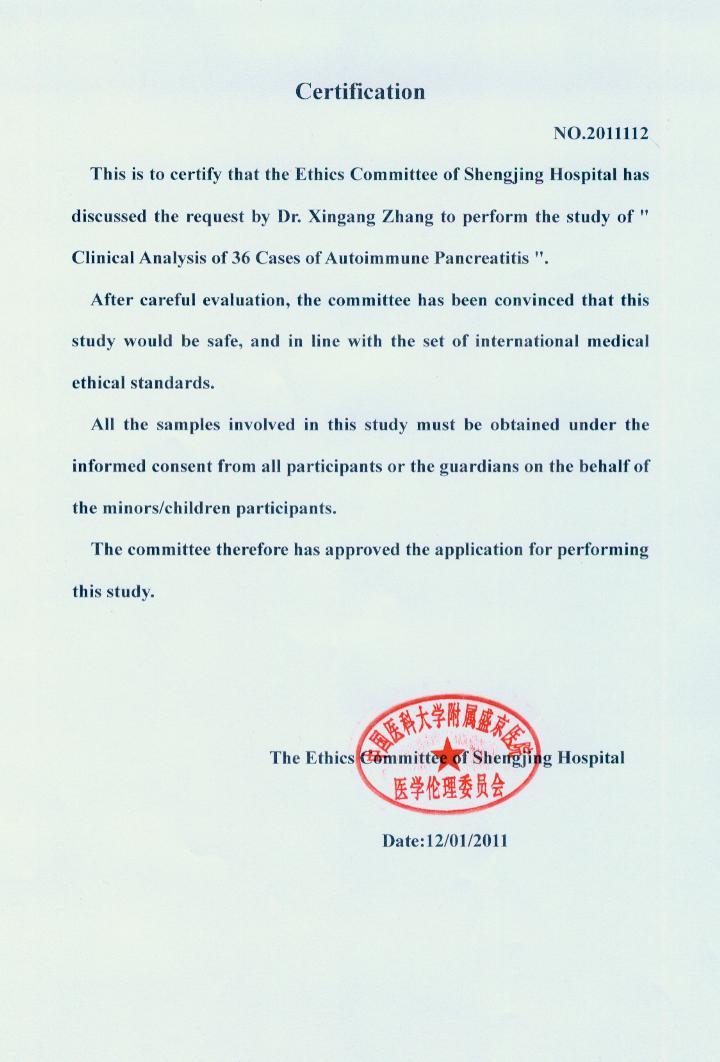

Supplement: Figure S1 — Ethics committee certification. (JPG) [file pone.0044808.s001.jpg]
